# Singing and music making: physiological responses across early to later stages of dementia

**DOI:** 10.12688/wellcomeopenres.16856.1

**Published:** 2021-06-14

**Authors:** Nina Walker, Sebastian J. Crutch, Julian West, Fergal W. Jones, Emilie V. Brotherhood, Emma Harding, Paul M. Camic

**Affiliations:** 1 Salomons Institute for Applied Psychology, Canterbury Christ Church University, Tunbridge Wells, Kent, UK; 2 Dementia Research Centre, Department of Neurodegeneration, Queen Square Institute of Neurology, University College London, London, UK; 3 Open Academy, The Royal Academy of Music, London, UK

**Keywords:** dementia, physiological measurement, video analysis, psychosocial activities, music, singing, wellbeing

## Abstract

**Background**: Music based interventions have been found to improve the wellbeing of people living with dementia. More recently there has been interest in physiological measures to provide additional information about how music and singing impact this population.

**Methods:** This multiple-case study design explored physiological responses (heart rate-HR, electrodermal activity-EDA, movement, and skin temperature-ST) of nine people with mild-to-moderate dementia during a singing group, and six people in the later stages of dementia during an interactive music group. The interactive music group was also video recorded to provide information about engagement. Data were analysed using simulation modelling analysis.

**Results**: The singing group showed an increase in EDA (p < 0.01 for 8/9 participants) and HR (p < 0.01 for 5/9 participants) as the session began. HR (p < 0.0001 for 5/9 participants) and ST (p < 0.0001 for 6/9 participants) increased during faster paced songs. EDA (p < 0.01 all), movement (p < 0.01 for 8/9 participants) and engagement were higher during an interactive music group compared to a control session (music listening). EDA (p < 0.0001 for 14/18 participants) and ST (p < 0.001 for 10/18 participants) increased and in contrast to the responses during singing, HR decreased as the sessions began (p < 0.002 for 9/18 participants). EDA was higher during slower music (p < 0.0001 for 13/18 participants), however this was less consistent in more interactive sessions than the control. There were no consistent changes in HR and movement responses during different styles of music.

**Conclusions**: Physiological measures may provide valuable information about the experiences of people with dementia participating in arts and other activities, particularly for those with verbal communication difficulties. Future research should consider using physiological measures with video-analysis and observational measures to explore further how engagement in specific activities, wellbeing and physiology interact.

## Introduction

Worldwide, about 50 million people have a form of dementia with about 10 million news cases being identified each year ( World Health Organisation (WHO), 2020). Within the United Kingdom (UK) there are an estimated 850,000 people currently living with dementia, and this is expected to rise to 1.6 million by 2040 ( Wittenberg *et al.*, 2020). Symptoms of dementia vary for each individual and type of dementia, affecting memory, thinking, behaviour and the ability to perform everyday tasks ( WHO, 2019). The National Institute for Health Care Excellence (NICE) has stated that available medications only offer small cognitive, functional and behavioural benefits for people with mild-to-moderate dementia ( NICE, 2019). Neuroleptic medications are often prescribed to manage the behavioural, psychological and social symptoms of dementia (BPSD) with some positive outcomes ( Kratz, 2017), yet these medications often have side effects and the evidence for the efficacy is mixed ( Bessey & Walaszek, 2019). Finding psychosocial interventions to improve the quality of life of people living with dementia (PLWD) and their carers is therefore warranted.

### Theories of wellbeing in dementia

Although historically, the primary focus of dementia care has been attending to physical care needs, there have been significant shifts towards considering the individual’s higher order needs, highlighted by the theory of “personhood” ( Kitwood, 1997). Personhood emphasises comfort, attachment, inclusion, occupation and identity as integral to wellbeing. Kitwood notes that care environments that do not foster these needs lead to a state of “illbeing” for the person with dementia. In recent years more consideration has been given to the wellbeing of the individual in the context of their relationships. Relational theories of dementia offer the opportunity to encapsulate the reciprocity and interdependence of caring relationships ( Clare *et al.*, 2020) and how these relate to the wellbeing of an individual. It has also been proposed that agency, an important theoretical concept linked to wellbeing for people living with dementia, can also be considered as relational ( Zeilig *et al.*, 2019). Nolan *et al.* (2004) proposed the “senses framework” which suggests that all parties involved in caring need to promote a sense of security, belonging, continuity, purpose, achievement and significance. These theoretical shifts in conjunction with the lack of pharmaceutical treatment have created an increased emphasis on the importance of psychosocial interventions to improve the wellbeing of PLWD and to develop relevant dementia-specific psychometric measures ( Strohmaier *et al.*, 2021).

### Psychosocial interventions and wellbeing

Psychosocial interventions incorporate a broad range of activities which share a common aim of improving quality of life. Effective interventions have been found to improve wellbeing in several ways. These include enabling the individual to maintain self-esteem and belonging ( Brod *et al.*, 1999). As with more traditional one-to-one therapy, both the content and the process may play a role in the intervention. Aside from the stimulation of the activity itself, other important factors may include interactions with others, physical movement and/or individual meaning of the activity ( Clare *et al.*, 2020). Maintaining relationships with people with a dementia diagnosis can feel challenging in the later stages. Interactions often become task-oriented due to the caregiver feeling solely responsible for initiating social interactions ( Penrod *et al.*, 2007). Paid carers may start to focus more on basic care needs when a PLWD is less able to respond during interactions ( Edvardsson *et al.*, 2014), particularly when they have not been trained to provide stimulating activities ( Mowrey *et al.*, 2013). Incorporating the aforementioned theories of wellbeing into the design and implementation of psychosocial interventions may be beneficial. For example, the fostering of personhood ( Kitwood, 1997) within an intervention may be achieved by ensuring the activity is personally meaningful and inclusive. Camic *et al.* (2013) proposed that Nolan’s five senses framework ( Nolan *et al.*, 2004) could be utilised as a way of theoretically understanding and evaluating psychosocial interventions for PLWD. Observing interactions within a group intervention that relate to security, belonging, continuity, purpose, achievement and significance may therefore provide information on how beneficial an intervention is for the person’s wellbeing.

### Musical interventions for people with dementia

The ability to recall and respond to music is often retained for longer than other information ( Cuddy & Duffin, 2005) and benefits related to cognition and wellbeing are well documented (e.g. Gallego & Garcia, 2017; Särkämö, 2018). Music-based activities have also been reported to reduce aggressive behaviour ( Clark *et al.*, 1998), stimulate communication ( Clare *et al.*, 2020) and are cost effective when compared to medication and increased levels of care ( Livingston *et al.*, 2014). A review by Van der Steen *et al.* (2018) however, concluded that quality of evidence is low and although music-based activities may improve depression, they have little or no impact on agitation or emotional wellbeing.

### Stress, emotion and physiological responses

The relationship between an individual’s emotional state and physiological responses is complex. The autonomic nervous system (ANS), which is made up of the parasympathetic (PNS) and sympathetic nervous systems (SNS) has a direct role in stress response with the SNS activating and creating the “fight or flight response”. Stress can therefore often be detected using physiological parameters that are influenced by SNS such as increased heart rate (HR) and electrodermal activity (EDA) ( Wijsman *et al.*, 2011). The ANS has been considered as integral to the emotional response of healthy individuals and linked to specific emotions ( Kreibig, 2010). Stemmler (2004) reported on a meta-analysis of autonomic responding in anger and fear and found considerable differences between the two, despite similar arousal characteristics. In contrast Barrett (2014) claimed that it is not possible to claim that emotion has “unique autonomic signatures” (p.41).

### Wellbeing and physiological responses during musical interventions

It is widely accepted that music has the capacity to alter emotions and research has shown healthy adults effectively using music to regulate how they are feeling ( Chen *et al.*, 2007; Getz *et al.*, 2014). Listening to music has been associated with arousal including increased EDA, HR and respiration rate ( Gomez & Danuser, 2004; Salimpoor *et al.*, 2009). It has also been found to lower arousal in the presence of stressors ( Thoma *et al.*, 2013). Faster tempo (over 120 bmp), staccato music is more likely to induce arousal including increased blood pressure, HR and skin conductance ( Bernardi *et al.*, 2006; Gomez & Danuser, 2007). Other factors including listening to music with a friend or self-selecting music have been suggested to increase positive emotional responses ( Liljeström *et al.*, 2013). There is emerging research measuring physiological responses in PLWD during psychosocial interventions ( Hsu, 2015; Suzuki *et al.*, 2007; Williams *et al.*, 2016). A review by Thomas *et al.* (2018) concluded that research concerning physiological interventions and music is limited in a dementia population, but studies measuring HR and heart rate variability (HRV) showed statistically significant changes within sessions. However, Raglio *et al.* (2010) found no significant longitudinal changes in HR over a music therapy intervention, suggesting the benefits may be limited to brief moments in time. Interpreting HR is not straightforward as it is impacted by a range of factors including movement, anxiety and excitement ( Wilhelm *et al.*, 2006), therefore measuring in conjunction with other information such as observations may be beneficial.

EDA is commonly used as a measure of arousal as it is considered a reliable marker of sympathetic activity ( Andreassi, 2007). An increase in EDA has been suggested to indicate agitation in PLWD as increases have been found to occur just before agitation can be visually observed ( Melander *et al.*, 2017). A review of the ANS activity in emotion linked increased EDA to fear and disgust but also to happiness and anticipatory pleasure in healthy adults suggesting it is difficult to make conclusions based on the physiology alone ( Kreibig, 2010). Acute stress has been associated with a short-term drop in skin temperature related to an increase in core temperature ( Oka *et al.*, 2001) and has therefore been suggested as a valuable non-invasive way of quantifying stress ( Herborn *et al.*, 2015). To date, no research has been identified observing changes in ST during music-based interventions for PLWD. There is also a sparsity of research on physiological responses in the later stages of dementia; this research may be particularly valuable for individuals that are often less able to communicate their experiences verbally and may not appear interested or engaged to observers.

### Rationale

The above research has outlined emerging evidence that physiological measures may be a helpful tool for understanding the experiences of PLWD during psychosocial interventions. Using individual case studies to take a more detailed look at individual experiences within smaller sections of an intervention may enable a richer understanding of what happens physiologically during musical interventions and how different responses relate to each other. Kitwood’s (1997) theory of personhood and the senses framework by Nolan *et al.* (2004) suggest that the beneficial aspects of an intervention may be in the sense of inclusion, achievement and purpose which could depend on interpersonal factors aside from the type of intervention. There is no research to date that we are aware of that considers how physiological responses relate to recorded observations during psychosocial interventions for this population. Observing how physiological responses relate to engagement and individual interactions may be a beneficial way of understanding more about the experiences of people with dementia during psychosocial interventions.

### Aims of the present study

This research consists of two linked studies using previously collected and unanalysed data from two music-based interventions for people at different stages of dementia. These studies aimed to gain a better understanding of what physiological responses might convey about their experiences, and how they may relate to wellbeing. This research also addresses National Health Service (NHS) values including “compassion” and “commitment to quality of care” as the activities are designed to alleviate distress and improve wellbeing for people with a dementia. Trying to understand and improve the activities for people in the later stages of dementia also fits with another NHS value that “everybody counts”, regardless of ability or health status ( NHS Constitution, n.d.). As previously stated, music has been linked in an increase in physiological arousal ( Gomez & Danuser, 2004; Salimpoor *et al.*, 2009). Physiological responses would therefore be expected to increase as the music begins, compared to baseline (H1, H5). Specific hypotheses have been formulated based on previous research ( Bourne *et al.*, 2019; Gomez & Danuser, 2007; Thomas *et al.*, 2018).

***Study 1 and study 2 hypotheses***

***Study 1***

H1: Physiological responses will be significantly higher during the first song compared to baseline

H2: Physiological responses will differ during faster paced and slower paced music

***Study 2***

H3: Physiological responses will be significantly higher during the intervention sessions (sessions 1 and 6) compared to a control session (music listening).

H4: There will be no significant difference between the physiological responses in the intervention sessions (session 1 and 6)

H5: Physiological responses will be significantly higher during the first song compared to baseline

H6: Physiological responses will differ during different types of music

H7: Changes in physiological responses will be associated with ratings of engagement and visible engagement from observations

H8: Peaks in physiological data will be associated with visible engagement

## Methods

This research consists of two linked mixed-methods multiple-case A-B design studies based on archival data from naturalistic settings ( Yin, 2003) and was part of the Created Out of Mind research programme at the Wellcome Collection ( Brotherhood *et al.*, 2017). Barlow *et al.* (2007) suggests that replication can be established with a minimum of four case studies and the design enables a more sensitive detection of change than group averages. Study 1 included nine case studies of the physiological responses of people with mild-to-moderate dementia during one session of a community singing group. Study 2 included six more detailed case studies, collating information on participants who had attended a control session and two intervention sessions of an interactive music group. These participants were in the later stages of dementia, living in a residential care home. The data were collected in autumn 2017 as part of the Created Out of Mind project at the Hub at Wellcome Collection, London.

### Materials used in both studies

Empatica-E4® sensor wristbands were worn by all participants and measured HR, EDA, movement (accelerometer (ACC)) and ST. The sensor produced a per-second numeric output related to each physiological measure, with differing sampling rates. EDA and ST produced four readings per second (4Hz), HR one reading (1Hz) and ACC 32 readings (32Hz). Audio recordings were made of both groups in order to compare physiological measures to the activity.

### Ethical procedures for both studies

Ethical procedures are reported below. Ethics approval was granted by Canterbury Christ Church University, Salomons Institute Ethics Panel (Study 1 approval number: 201516; Study 2 approval number: 201617). The studies adhered to British Psychological Society (BPS) ethical guidelines (2014) and those of Research Ethics Service of the Health Research Authority ( HRA, 2019). There were no reports from participants or observations by staff or researchers of distress during any of aspect of the sessions. During and after the sessions, no reports of discomfort or desire to remove the wristband were voiced by participants nor observed by researchers or accompanying staff; no participants withdrew from the study. Data were encrypted and stored anonymously using participant ID numbers and saved on a password protected hard drive. All data were stored in accordance with the Data Protection Act (2018). Following the interactive music sessions, video data were downloaded onto an encrypted and password protected file by one of the researchers. Video data were only viewed using an encrypted hard drive and the data were downloaded to password-protected computers in secure, non-public locations. Consent was considered for each individual as required by the mental capacity act (MCA, Department of Health, 2005).

***Study 1***

All participants were deemed to have capacity to consent. Participants were informed about the research through a question and answer session, given a participant information sheet to consider for a week beforehand, provided time for individual discussion the following week, and only then written informed consent was taken.

***Study 2***

In study 2, none of the participants were deemed able to give consent due to cognitive impairment associated with advanced stages of dementia; this was determined jointly by researchers and residential care management. Family members who were legal guardians were therefore invited to a group information meeting at the residential care home, where the study was explained and questions answered. They were provided written information about the study and asked to consider, over the course of seven days, whether they wanted their family member to participate; all agreed to allow participation. As part of the consent process and following HRA guidance, family members acted as “consultees” and were asked to agree to the following statement “If my relative had been able to give consent for this I believe they would have agreed to participate and think this is something they would have wanted.” Consultees were also asked to agree to provide their input each week to assess whether they believed their family member wanted to continue to participate; all agreed to ongoing participation. Musicians and staff members signed consent forms to participate in the research and to be video and audio recorded. Staff members volunteered to participate and were clearly informed by residential care management that they were under no obligation to participate; their participation became part of their care duties during the study.

### Study 1

***Participants.*** Using convivence sampling, participants were recruited from an existing singing group for PLWD and their carers. The organisation hosting the singing group was first approached to take part by JW and PC. After the organisation’s agreement, all group members were invited to take part and inclusion criteria were purposely kept broad: a diagnosis of mild-to-moderate dementia and ability to give informed consent.

***Procedure.*** Empatica-E4 were fitted to participants’ wrists on their dominant hands. The session ran for approximately one hour and was led by an experienced choral conductor with an accompanying pianist. It consisted of a welcome song, stretching and vocal exercises, followed by four songs with slower paced and faster paced tempos: Bella Mama (a Torres Strait Islands song, 90 bpm ^1^), Bei Männern (Mozart, from the Magic Flute, 86 bpm), The Lion Sleeps Tonight (Wimoweh) (South African, 126 bpm), and The Erie Canal (American popular, 126 bpm) broken down and practiced and then sung in their entirety. Participants were intermittently asked to stand and sit down. The style of songs ranged from a legato and slower paced style to a quicker and staccato style. Two songs from the four selected, differing in style, were used for comparison ( Table 1). Following the session, participants returned to their tables for refreshments and removal of their wristbands. Versions of these songs are also freely available on YouTube.

**Table 1.** Musical styles for comparison.

	Style	Description
Bei Mannern (Slower)	Classical	Major key, crescendo and diminuendo, Adante (walking pace)
Eerie Canal (Faster)	Show tune	Major key, Forte (loud), Energetic

The group had been running for approximately two months. The participants appeared comfortable with the environment and group, reducing the likelihood of confounding variables such as anxiety about singing, socializing with unknown people, and not knowing the facilitator, thus increasing the validity of the data. Although there was no control group, data collected immediately before the singing began was used as a baseline.

***Data analysis.*** All participants were included in the analysis. Audio recordings were matched to the timestamped pre-collected physiological measures to determine the time in the session. Data sets were then collated for all timeframes and physiological measures for each individual case study ( Table 2). Physiological responses were chunked into ten second intervals and then analysed using the Simulation Modelling Analysis (SMA) program (version 07.30.20) which enables case-based time-series studies with multiple observations to determine individual change ( Borckardt & Nash, 2014). Non-parametric tests (Spearman’s rho) were administered due to the small sample size. Bonferroni corrections were used to control for multiple comparisons by dividing significance of 0.05 by the number of tests administered (72). 

**Table 2.** Data selection in study 1.

Data sets		Length of data set
1	Pre music beginning	2m
2	First song of session	2m 25s
3	After first song	1m 44s
4	Energetic (fast) music	3m 22s
5	Adante (walking pace) music	2m 45s

### Study 2

The case studies in study 1 provided useful information about physiological responses during a singing group and how musical style might play a role in wellbeing during mild-to-moderate stages of dementia. These data were interpreted with the knowledge that the group was popular and voluntarily attended, however this raised questions around how physiological responses might differ in the later stages of dementia and how these could be interpreted in a population that is not able to give consent to an intervention or necessarily verbally communicate their experiences.

***Participants.*** A residential care home caring for people at advanced stages of dementia was approached by JW and PC and invited to take part in the study. After agreement was obtained, senior care home staff decided what residents would be able to attend the sessions over an 8-week period. This is a process the organisation routinely engages in when considering appropriate activities for residents. Six residents were chosen and family members, who were legal guardians, were informed about the study in writing and invited to attend a routinely scheduled monthly meeting of staff and family members for further discussion, to answer any questions and sign consent forms. Recruitment criteria included the following: (i) a confirmed diagnosis of dementia; (ii) Clinical Dementia Rating Scale ( Morris *et al.*, 1997) score of 2–3 (advanced) as rated by care staff; (iii) aged 60 or above; and (iv) able to sit in a room for an hour in a group setting. PLWD that had (i) a clinical dementia rating of below 2; (ii) significant hearing difficulties that cannot be corrected, even with a hearing aid; or (iii) disruptive behaviour during group activities in the care facility (e.g. aggressive behaviour) were excluded. These criteria were screened by care staff at the care home and verified by one of the researchers.

***Procedure.*** The interactive music group modelled on Music for Life, ran for eight 1 hour-long sessions at the same time every week. To minimise any potential burden on participants, data were only collected in the control session and intervention sessions 1 and 6. Session 1 was chosen because it was the beginning of the intervention, and session 6 chosen because it was a time point well into the activity but not the final session, which was session 8. Music for Life was founded in 1993 by Linda Rose and brings together professional musicians, people living with dementia and those that care for them to explore the benefits of music making together. A large collection of percussion instruments was accessible for all group members, and the music making is entirely improvisatory in nature, with musicians responding to sounds, words and gestures contributed by other participants. The music making provides a context within which each person’s contribution can be heard and valued equally, and enables ‘in the moment’ creative collaborations and interactions between everyone involved. A week prior to the group starting, a control session took place in the same room and time of day. The control involved listening to recorded music of a similar style to that in the intervention that was played by the same musicians. Participants were asked to wear the Empatica-E4 wristbands during the control session, the first session and session six. Musicians and two researchers were also present at this session in order to create similar conditions to the intervention sessions. The intervention sessions consisted of three main pieces of music with additional improvised music interspersed. Musical styles ranged from slower tempo, quieter music to upbeat, staccato forte music. Instruments included a harp, flute, bongo drums and a range of handheld percussion instruments that participants were encouraged to use by staff and musician-facilitators.

***Materials.*** In addition to audio recording, a Fly 4K 360-degree camera™ was used to video record the group in order to capture interactive components and processes for each individual as clearly as possible with minimal intrusion. The Video Coding – Incorporating Observed Emotion (VC-10E) scale was used to monitor engagement from the video footage. This measure was chosen as it is designed specifically for video analysis ( Jones *et al.*, 2015) and provides information about the nature of the engagement (positive or negative) in addition to absence or presence of engagement. Inter-rater reliability has been found to be exceptionally high across ten different video coders (95.25%) when comparing within a 1 second tolerance interval. An optimal inter-rater reliability of 95% has also been obtained across dependent measures.

***Data analysis.*** Datasets relating to each of the pre-determined measures of interest were collated and analysed ( Table 3).

**Table 3.** Data selection in study 2.

Measure	Session	Data analysis	Length of data set
1	Control	Pre music beginning	2m
2	Control	First piece of music	5m 9s
3	Control	Welcome song comparison	5m 23s
4	Control	Whole session	55m 46s
5	Control	Fast music	3m 45s
6	Control	Slow music	4m 12s
7	Session 1	Pre music beginning	2m
8	Session 1	First piece of music	5m 33s
9	Session 1	Welcome song	5m 20s
10	Session 1	Whole session	62m 11s
11	Session 1	Fast music	5m 35s
12	Session 1	Slow music	3m 35s
13	Session 6	Pre music beginning	2m
14	Session 6	First piece of music	5m 21s
15	Session 6	Welcome song	8m 17s
16	Session 6	Whole session	58m 16s
17	Session 6	Fast music	3m 34s
18	Session 6	Slow music	4m 15s

In a similar approach to study 1, physiological responses were chunked into ten second intervals and then analysed using SMA as time-series data. In order to determine how the participants’ presentation related to the physiological measures, engagement during fast and slow music was rated for three participants in three sessions using the VC-10E. This involved rating the number of seconds that categories of positive and negative engagement were present. Participants were selected that were visibly different in their level of engagement and to include male and female participants. Engagement was rated once by an independent clinician who was not aware of the research hypothesis. Points of increased physiological activity were also identified by sorting the physiological measures from greatest to smallest. The time periods with increased physiological activity were then observed in the video to record individual activity and context. This was only possible to undertake for HR, EDA and ST due to the number of readings per second.

## Results

### Study 1

Study 1 consists of 9 individual case studies of people in early-to-middle-stage dementia where physiological data were collected throughout the same singing session ( Walker *et al.*, 2021a) ( Table 4). All singing group members were deemed appropriate to participate. Twelve people were approached and 1 declined without providing a reason. Physiological data for 2 participants were not sufficiently recorded by the Empatica-E4 and not included in the analysis. Hypothesis 1 (H1) states that physiological measures will be significantly higher during the first song than during baseline and Hypothesis 2 (H2) states that physiological responses will differ during fast and slower paced music. Results are reported by each physiological measure in turn. The descriptive statistics and the differences between measures for during different conditions in Study 1 are presented in Table 5.

**Table 4.** Characteristics of study 1 participants.

Par number	Diagnosis	Age	Gender	Ethnicity
1	AD	75–80	M	White British
2	Mixed AD/FTD	75–80	M	White British
3	AD	80–85	M	White British
4	AD	70–75	F	White European
5	AD	> 85	F	White European
6	DLB	65–70	M	White British
7	AD	75–80	M	White British
8	FTD	65–70	M	White European
9	AD	> 85	F	White British

AD = Alzheimer’s disease, FTD = Frontotemporal dementia, DLB = Dementia with Lewy bodies

**Table 5.** Descriptive statistics and significance results comparing physiological responses during the first song to baseline and during fast and slow music (study 1).

Measure	ID	Baseline		First song		Fast		Slow		Base- First		Fast- Slow	
		M	SD	M	SD	M	SD	M	SD	Rho	p	Rho	p
HR	P1	104.43	7.03	88.41	14.34	90.01	11.02	70.32	9.52	-0.49	.0001 **	-0.7	.0001 **
	P2	70.62	2.21	79.14	11.17	74.35	3.99	74.48	4.05	0.4	.008 *	0.06	0.367
	P3	78.49	2.94	104.62	11.23	87.92	4.64	85.00	7.30	0.77	.0001 **	-0.17	0.134
	P4	63.62	2.52	69.43	9.08	56.27	2.79	58.39	5.13	0.23	.08	0.17	0.151
	P5	68.67	9.22	79.55	6.03	83.99	13.81	84.17	9.10	0.5	.0001 **	0.05	0.4
	P6	69.92	7.73	79.64	5.94	94.22	8.89	78.34	5.34	0.565	.0001 **	-0.755	0.0001 **
	P7	97.40	0.96	95.68	7.40	76.83	5.05	62.61	5.28	0.0239	0.472	-0.78	.0001 **
	P8	59.74	1.12	77.22	7.48	77.43	10.10	64.98	5.34	0.76	.0001 **	-0.5864	.0001 **
	P9	82.73	5.29	79.57	5.22	80.10	2.17	74.82	1.15	-0.24	.067	-0.83	.0001 **
EDA ^2^	P1	0.34	0.39	4.49	3.95	2.86	1.41	3.30	1.77	0.55	.001 *	0.3	.045 *
	P2	0.12	0.00	0.13	0.00	0.20	0.02	0.25	0.07	0.8	.0001 **	0.37	.009 *
	P3	0.20	0.18	1.35	0.34	3.28	0.52	1.81	0.53	0.78	.0001 **	-0.86	.0001 **
	P4	0.12	0.00	0.14	0.01	0.21	0.01	0.10	0.01	0.75	.0001 **	-0.86	.0001 **
	P5	0.10	0.06	0.89	0.05	0.01	0.00	1.02	0.29	0.73	.0001 **	0.86	.0001 **
	P6	0.14	0.00	0.17	0.02	0.36	0.09	0.16	0.00	0.67	0.0001 **	-0.861	0.0001 **
	P7	N/A	N/A		0.71	0.19	1.29	0.38		N/A	N/A	0.73	.0001 **
	P8	4.38	0.36	6.38	0.28	3.39	0.62	4.70	0.63	0.79	.0001 **	0.71	.0001 **
	P9	0.35	0.01	0.40	0.05	0.14	0.01	0.15	0.01	0.47	.001 *	0.22	0.093
ACC ^3^	P1	1.01	0.02	1.05	0.00	0.99	0.01	1.00	0.01	0.4	.006 *	0.15	0.185
	P2	0.99	0.00	0.99	0.00	1.00	0.02	0.99	0.01	-0.7	.0001 **	-0.08	0.315
	P3	0.99	0.01	1.10	0.09	1.02	0.01	1.01	0.01	0.7	.0001 **	-0.01	0.459
	P4	0.90	0.03	0.75	0.16	0.93	0.08	0.84	0.08	-0.45	.001 *	-0.49	.001 *
	P5	0.98	0.01	1.00	0.19	0.99	0.00	0.98	0.01	0.4	.001 *	-0.27	0.06
	P6	1.35	0.01	1.02	0.13	1.18	0.02	0.16	0.00	-0.78	.0001 **	-0.855	0.0001 **
	P7	0.99	0.00	0.10	0.00	1.00	0.00	1.00	0.00	0.78	.0001 **	0.43	0.005
	P8	0.98	0.00	1.01	0.03	0.99	0.01	0.99	0.00	0.76	.0001 **	-0.06	0.347
	P9	1.01	0.01	1.02	0.01	1.00	0.01	1.02	0.00	0.31	.018 *	0.79	.0001 **
ST ^4^	P1	31.23	4.75	33.34	0.48	33.16	0.04	32.77	0.03	-0.08	0.307	-0.862	.0001 **
	P2	30.34	0.03	29.96	0.30	29.80	0.18	29.74	0.09	-0.7	.0001 **	-0.21	0.099
	P3	32.57	0.33	32.43	0.30	31.87	0.08	31.84	0.13	-0.22	0.067	-0.14	0.177
	P4	28.35	0.04	27.96	0.14	31.93	0.06	28.87	0.05	-0.78	.0001 **	-0.86	.0001 **
	P5	33.16	0.34	33.09	0.31	32.96	0.08	33.28	0.07	-0.1	0.29	0.86	.0001 **
	P6	28.07	0.05	28.50	0.12	31.01	0.03	29.25	0.15	0.78	.0001 **	-0.864	0.0001 **
	P7	22.55	0.20	23.48	0.92	32.39	0.16	31.94	0.02	0.63	.0001 **	-0.78	.0001 **
	P8	32.85	0.13	33.21	0.18	34.60	0.05	34.04	0.06	0.7	.0001 **	-0.86	.0001 **
	P9	30.33	0.09	30.31	0.08	30.63	0.02	30.39	0.04	-0.17	0.158	-0.86	.0001 **

^1^heart rate, ^2^electrodermal activity, ^3^movement, ^4^skin temperature. Colour code: dark colours indicate significant differences after Bonferroni correction (**p<0.0007); pale colours indicate uncorrected standard threshold (*p<0.05). Green = higher during first song than baseline; red = lower during first song than baseline; blue = higher during slow than fast music; orange = lower during slow than fast music.

***HR.*** Supporting H1, HR was significantly higher (p < 0.008) during the first song compared to baseline for five participants. Only one participant (P1S1) had a significantly higher HR at baseline (p < 0.001) compared to during the first song ( Table 6).

**Table 6.** Characteristics of study 2 participants.

Participant	Diagnosis	Age	Gender	Ethnicity
1	Atypical/mixed	97	Female	White British
2	AD	93	Female	White British
3	Mixed AD/VaD	92	Male	White British
4	AD	92	Male	White British
5	AD	85	Male	White British
6	VaD	88	Female	White British

AD = Alzheimer’s disease, VaD = Vascular dementia

The HR of five participants (P121, P6S1, P7S1, P8S1, P9S1) was significantly higher (p < 0.0001) during the faster paced music compared to the slower paced music in support of H2. There was no significant difference in HR during different styles of music for the four remaining participants.

***EDA.*** EDA was higher during the first song compared to baseline for eight of the nine participants (p < 0.001) supporting H1. Data was not collected for the remaining participant (P7S1). EDA during different styles of music were more mixed, therefore H2 was not supported. EDA of five participants (P1S1, P2S1, P5S1, P8S1, P9S1) was significantly higher (p < 0.05) during the slower paced music and the EDA for three participants (P3S1, P4S1, P6S1) was significantly higher during faster paced music (p < 0.0001).

***Movement.*** Supporting H1, movement was significantly higher during the first song compared to baseline for six participants (p < 0.05). However, movement was significantly higher (p < 0.001) at baseline for three participants (P2S1, P4S1, P6S1). There were only three significant differences between the level of movement during the fast (p < 0.001) and slow music (p<0.0001) and the results were mixed, therefore H2 was not supported.

***Skin temperature.*** Skin temperature was significantly higher (p < 0.0001) during the first song than at baseline for three participants (P6S1, P7S1, P8S1) and higher during baseline (p < 0.0001) for two participants (P2S1, P4S1), therefore H1 is not supported. Skin temperature was significantly higher (p < 0.0001) during the fast music for six participants supporting H2. Skin temperature was only significantly higher (p < 0.0001) during the slow music for one participant (P5S1).

***Summary of physiological data for study 1.*** H1 stated that physiological measures will be significantly higher during the first song than before the session began. The results summarizing the data from the case studies ( Table 5) indicate that there was an overall increase in physiological measures during the first song compared to baseline, therefore H1 is partially supported. Amongst all nine case studies there were sixteen significantly higher results during the first song, compared to only four significantly lower responses. The most consistent change from baseline was a significant increase in EDA for seven of the eight participants with EDA recordings. Only one person had a decrease in HR during the first song and the data suggests a common pattern of HR increasing during the first half of the first song, then decreasing. The differences between ST and movement before and during the first song were mixed. Movement was higher during the first song for most participants, however three participants moved significantly less.

H2 stated that there will be a significant difference between physiological measures during different styles of music. As can be seen in Table 5, the collated results are mixed. Overall, there were more robustly significant differences than not significant results which was consistent with H2, however some responses were significantly higher during the faster music and some were significantly higher during the slower music. HR was significantly higher during the faster paced song than the slower paced for five participants and there were no contrasting results. ST was significantly higher during the faster song for six participants and during the slower for only one participant. EDA and movement showed mixed results that did not support H2.

### Study 2

This study consisted of 6 participants ( Table 6) where the same physiological data were collected during a control session and two intervention sessions of an interactive music group, but with people with more severe dementia ( Walker *et al.*, 2021a). H3 and H4 were addressed in the first section as these hypotheses consider physiological measures across sessions. H5, H6 and H7 are then addressed in a subsequent section, considering physiological changes within the sessions. The final section addresses H8 by observing peaks in the data and how these relate to visible engagement.

**Figure 1.**
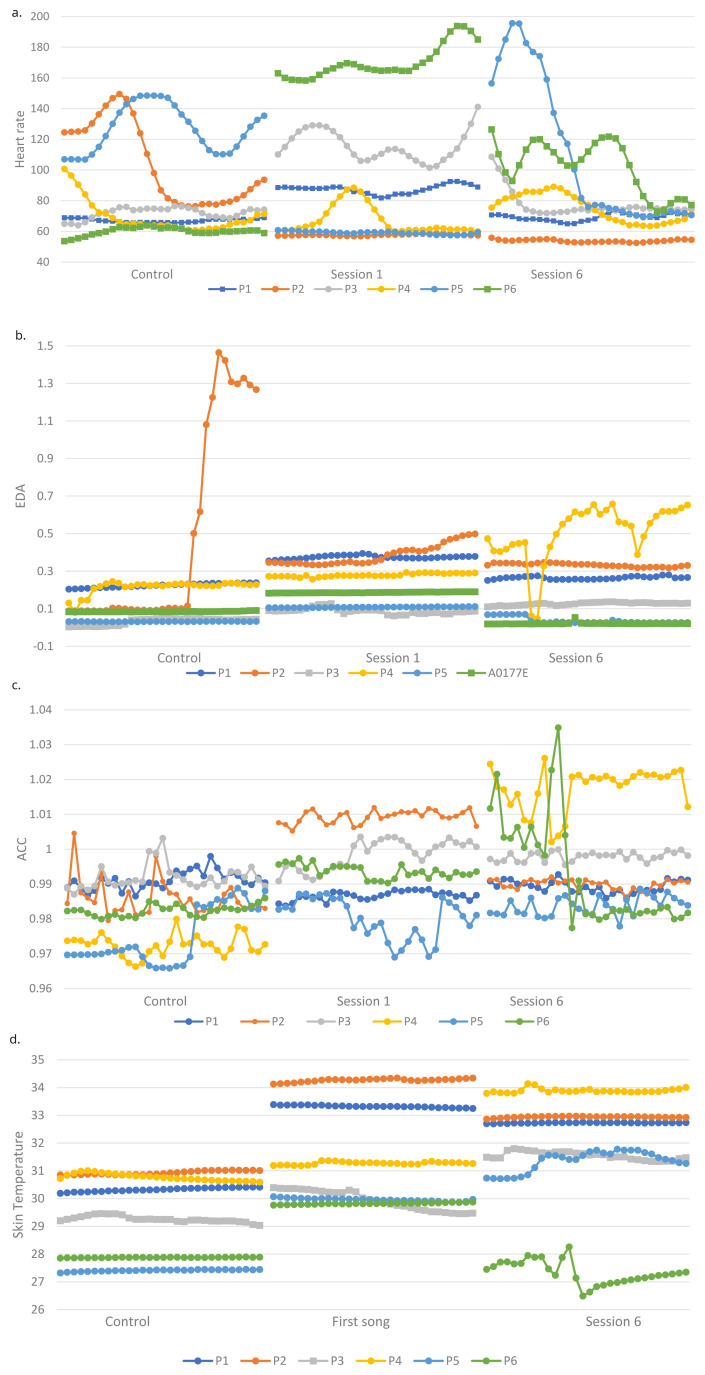
**a**. Heart rate (HR) of all participants during first song of control and two intervention sessions. **b**. Electrodermal activity (EDA) of all participants during first song of control and two intervention sessions. **c**. Accelerometer data (ACC) of all participants during first song of control and two intervention sessions. **d**. Skin temperature (ST) of all participants during first song of control and two intervention sessions.

***Changes in physiological measures across sessions.*** Figure 1 shows the physiological outcomes of participants over the sessions.

**HR.** H3 stated that physiological responses during the intervention sessions will be significantly higher than during a control session. The HR of three participants was significantly higher (p < 0.0001) during session 1 (P1S2, P3S2, P6S2) and session 6 (p < 0.001) (P1S2, P4S2, P6S2) compared to the control ( Table 7). In contrast, the HR of two participants were significantly higher (p < 0.0001) during the control than session 1 (P2S2, P4S2, P5S2), therefore H3 is not supported regarding HR. H4 stated that there will be no significant difference between physiological responses during the two intervention sessions. Four participants had a higher HR during session 1 than session 6 (p < 0.0001) and two participants had a higher HR during session 6 (p < 0.0001), therefore H4 was not supported.

**Table 7.** Descriptive statistics and significance results comparing physiological responses during the control session to intervention sessions.

Measure	ID	Control		Session 1		Session 6		Con-Session1		Con-Session6		Session 1–6	
		M	SD	M	SD	M	SD	Rho	Sig	Rho	Sig	Rho	Sig
HR ^1^	P1	67.09	1.17	87.51	2.76	69.33	2.23	0.87	.0001 **	0.5	.0001 **	-0.87	.0001 **
	P2	104.21	26.26	57.7	0.52	53.84	0.83	-0.87	.0001 **	-0.87	.0001 **	-0.87	.0001 **
	P3	72.06	3.55	115.81	9.84	77.26	8.53	0.87	.0001 **	0.33	.003 *	-0.85	.0001 **
	P4	69.24	10.27	67.03	9.14	75.1	8.92	-0.275	.017 *	0.38	.001 **	0.5	.0001 **
	P5	126.81	14.97	59.11	0.93	110.79	47.13	-0.87	.0001 **	-0.26	.033 *	0.61	.0001 **
	P6	60.04	2.51	170.15	10.64	101.91	16.53	0.87	.0001 **	0.87	.001 **	-0.87	.0001 **
EDA ^2^	P1	0.224	0.011	0.374	0.01	0.265	0.009	0.87	.0001 **	0.87	.0001 **	-0.87	.0001 **
	P2	0.448	0.539	0.367	0.035	0.333	0.009	0.29	.007 *	0.33	.003 *	-0.29	0.016
	P3	0.032	0.018	0.089	0.017	0.013	0.008	0.87	.0001 **	0.87	.0001 **	-0.8	.001 **
	P4	0.214	0.037	0.277	0.008	0.495	0.147	0.87	.001 *	0.75	.0001 **	0.75	.0001 **
	P5	0.031	0.001	0.108	0.002	0.018	0.018	0.87	.0001 **	-0.3	.01	-0.87	.0001 **
	P6	0.085	0.002	0.186	0.002	0.021	0.006	0.87	.0001 **	-0.87	.0001 **	-0.87	0.0001
ACC ^3^	P1	0.991	0.003	0.987	0.001	0.989	0.002	-0.77	0.0001 **	-0.37	.001	0.65	0.001 **
	P2	0.986	0.005	1.009	0.002	0.99	0.001	0.8661	0.0001 **	0.62	.0001 **	-0.8661	0.0001 **
	P3	0.992	0.003	0.998	0.004	0.998	0.001	0.6871	0.0001 **	0.72	.0001 **	-0.15	0.112
	P4	0.972	0.003	No data	No data	1.017	0.006	No data	No data	0.87	.0001 **	No data	No data
	P5	0.975	0.008	0.98	0.006	0.984	0.003	0.32	0.01	0.43	.0001 **	0.3	0.008
	P6	0.982	0.002	0.994	0.002	0.993	0.015	0.87	0.0001 **	0.22	0.047	-0.17	0.106
ST ^4^	P1	30.32	0.07	33.32	0.04	32.72	0.01	0.87	.0001 **	0.86	.0001 **	-0.86	.0001 **
	P2	30.93	0.07	34.25	0.06	32.94	0.03	0.86	.0001 **	0.86	.0001 **	-0.86	.0001 **
	P3	29.26	0.01	30.1	0.26	31.61	0.11	0.86	.0001 **	0.86	.0001 **	0.86	.0001 **
	P4	30.77	0.12	31.26	0.06	33.88	0.08	0.86	.0001 **	0.86	.0001 **	0.86	.0001 **
	P5	27.41	0.03	29.96	0.04	31.3	0.4	0.86	.0001 **	0.86	.0001 **	0.86	.0001 **
	P6	27.88	0.01	29.81	0.02	27.38	0.47	0.86	.0001 **	-0.53	.0001 **	-0.86	.0001 **

^1^heart rate, ^2^electrodermal activity, ^3^movement, ^4^skin temperature. Colour code: dark colours indicate significant differences after Bonferroni correction ( **p<0.0027); pale colours indicate uncorrected standard threshold (*p<0.05). Green = higher during first intervention session than control; red = higher during the control session than intervention session; brown = higher during session 1 than session 6; blue = higher during session 6 than session 1

**EDA.** Consistent with H3, six participants had significantly higher EDA during the first session compared to the control (p < 0.01), there were four robustly significant differences for two (p < 0.0001). Five participants had significantly higher (p < 0.01) EDA during session six than the control. P1S2, P3S2 and P5S2 had significantly higher EDA during session 1 than 6 (p < 0.001), whilst the opposite was true for P4S2 (p < 0.0001). H4 was therefore not supported.

**Movement.** Overall, there was more movement in the intervention sessions compared to the control in line with H3. Three of five participants showed more movement during the first session (p < 0.0001) and four participants showed more movement during session 6 than the control (p < 0.0001). There was only one contrasting result who moved more during the control than session 1 (p < 0.0001) (P1S2). Consistent with H4, only two of five participants showed significant differences in movement between the two intervention sessions, one participant moved significantly more (p < 0.0001) in session 6 (P1S2) and the other moved more (p < 0.001) in session 1 (P2S2).

**Skin temperature.** Supporting H3, ST was higher during the intervention sessions than the control for all participants (p < 0.0001) except P6S2 who had a lower ST in session 6 than the control (p < 0.0001). H4 was not supported, as three participants had a higher ST in session 1 than six (p < 0.0001) and the remaining three had the opposite response (p < 0.0001). 

**Figure 2.**
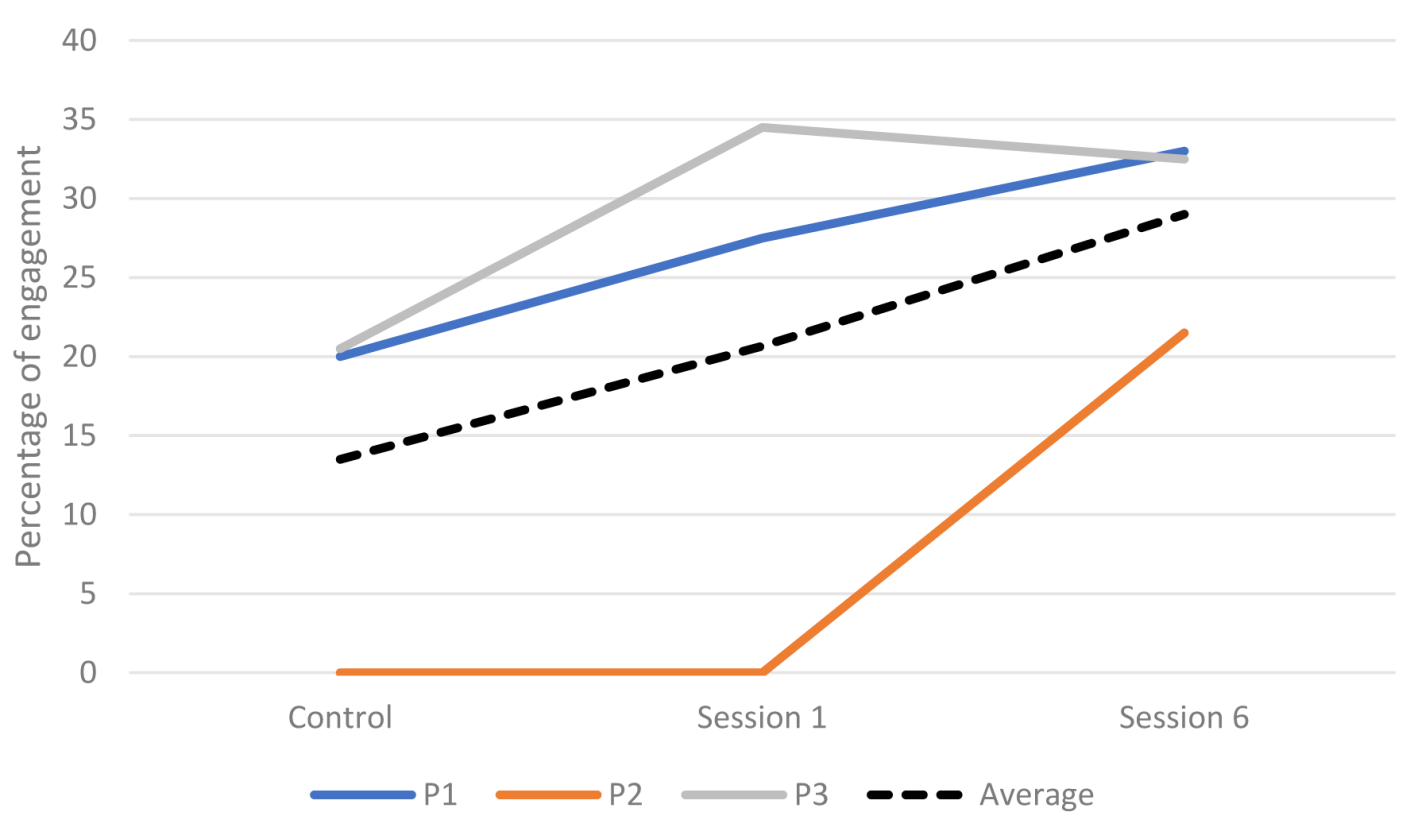
Percentage of engagement during the control and intervention sessions.

**Engagement across sessions.** The engagement of three participants was rated during faster paced and slower paced music of each session. The percentage of engagement for each participant can be seen in Table 8. There was an overall increase in engagement as the sessions progressed with the highest rated engagement occurring in session 6 ( Figure 2).

**Table 8.** Percentage of engagement during fast and slow music in the control and intervention sessions.

	Control		Session 1		Session 6	
	Positive	Negative	Positive	Negative	Positive	Negative
P1	20%	0%	27.5%	0%	33%	0%
P2	0%	0%	0%	0%	21.5%	0%
P3	20.5%	0%	34.5%	0%	32.5%	0%
Total	40.5	0	62	0	87	0

***Summary of physiological measures across sessions.*** Overall, physiological measures did appear to be elevated during the intervention sessions compared to the control session, which is consistent with H3 ( Table 7). EDA, movement and ST were more consistently higher during the intervention sessions whilst HR results were more mixed. Two participants’ responses were significantly higher across all four measures. There was a significant difference between measures during the intervention sessions with more being significantly higher during session 1 than 6, therefore H4 was not supported. HR was higher for four participants and EDA was higher for three participants in session 1. The average engagement was higher during the intervention sessions than the control session. All participants showed an increase in engagement as the intervention progressed.

***Physiological changes within the session***

**Heart Rate.** H5 stated that physiological responses would be higher during the first song than baseline. This hypothesis was not supported in the control condition, as the HR of four of the six participants was significantly lower (p < 0.001) once the song session began (P1S2, P2S2, P4S2, P6S2). Changes in HR as the session began were more mixed in the intervention sessions, with the HR of two participants being significantly higher (p < 0.005) and two significantly lower (p < 0.005) as the session began in both session 1 and session 6. This mixed picture of changes does not support H5.

H6 stated that physiological responses will differ significantly during fast and slow music. In the control session, differences in HR during fast and slow music were mixed. Three participants (P2S2, P3S2, P5S2) had significantly higher HR (p < 0.001) during the fast music and two participants (P1S2, P6S2) had significantly higher HR during the slow music(P < 0.05). During session 1, the HR of two participants was significantly higher (p < 0.0001) during the slow music (P4S2, P5S2) and one was significantly higher (p < 0.0001) during the fast music (P1S2). Differences in HR during session 6 were more aligned, with the HR of four participants being faster during the slow music (p < 0.05) (P1S2, P2S2, P4S2, P5S2), and only one participant being faster during the fast music (p < 0.0001) (P6S2).

**EDA.** In support of H5, EDA was significantly higher (p < 0.05) during the first song compared to baseline for most participants in the control and two intervention sessions. In the control session and session 1, EDA was higher for five participants during the first song (P1S2, P2S2, P3S2, P4S2, P6S2). EDA was higher for four participants during the first session (P1S2, P3S2, P4S2, P5S2, P6S2) and for four participants in session 6 (P1S2, P2S2, P3S2, P4S2). EDA was only significantly higher during baseline (p < 0.005) for one participant across all three sessions, in session 6 (P5S2).

Supporting H6, EDA was significantly higher during the slow music than the fast music for all participants during the control session (p < 0.0001). In session 1, EDA was higher for four participants during the slow music (p < 0.0001) (P3S2, P4S2, P5S2, P6S2), and one participant during the fast music (P1S2). In session 6, EDA was higher during the slow music for four participants (p < 0.05) (P2S2, P3S2, P4S2, P6S2) and during the fast music for two participants (p < 0.0001) (P1S2, P5S2).

**Movement.** The movement results did not support H5. In the control session, one participant demonstrated significantly more movement (p < 0.0001) during the first song (P1S2) and one participant demonstrated slightly more movement (p < 0.0001) during baseline (P6S2). In session 1, movement was slightly higher (p < 0.05) for one participant as the session (P3S2) began and slightly lower (p < 0.05) for two participants (P5S2, P6S2). In session 6, movement was significantly higher (p < 0.05) for one participant during the first song (P4S2) and significantly higher (p < 0.05) during baseline for two participants (P1S2, P3S2).

In partial support of H6, three participants of the control session (P2S2, P3S2, P5S2) and session 6 (P3S2, P4S2, P5S2) moved more during the fast music (p < 0.0001). The results from session 1 did not support the hypothesis as there was little difference in movement during the different types of music. Only three participants across all three sessions moved more during the slow music (p < 0.05).

**Skin temperature.** In support of H5, skin temperature was significantly higher (p < 0.05) for four participants during the first song compared to baseline during the control session (P1S2, PP4S2, P5S2, P6S2). Results were more mixed in session 1, with skin temperature being significantly higher (p < 0.0001) during the first song for two people P2S2, P6S2) and significantly higher (p < 0.05) at baseline for three participants (P1S2, P3S2, P5S2). In session 6, four participants had significantly higher (p < 0.0001) skin temperature during the first song (P1S2, P2S2, P3S2, P5S2) compared to only one participant who had higher skin temperature at baseline (p < 0.05) (P6S2).

In support of H6, skin temperature was significantly higher (p < 0.001) during the slow music for five participants in the control session (P2S2, P3S2, P4S2, P5S2, P6S2) and all participants in session 6 (p < 0.001). Results during session 1 were more mixed, with skin temperature being significantly higher (p < 0.0001) during the fast music for four people (P1S2, P3S2, P5S2, P6S2) and higher during the slow music for one person (p < 0.0001).

***Summary of physiological changes within the session.*** H5 was partially supported by an increase in some physiological measures during the first song compared to baseline, particularly EDA and ST ( Table 9). EDA was significantly higher during the first song for three participants of the control, five during session 1, and four during session six. ST was significantly higher for four participants during the first session, two of which also showed significantly higher EDA and significantly lower HR (P1S2, P6S2). EDA and ST were significantly higher for P6S2 in both the control and session 1, however there was no significant difference in session 6, suggesting changes as the intervention progresses.

**Table 9.** Descriptive statistics and significance results comparing physiological responses during the first song to baseline and during fast and slow music (study 2).

Measure	Session	ID	Baseline		First song		Fast		Slow		Baseline – first song		Fast - slow	
			M	SD	M	SD	M	SD	M	SD	Rho	p	Rho	p
HR ^1^	Control	P1	79.2	12.53	67.15	1.2	66.89	0.62	68.36	0.8	-0.73	.0001 **	0.69	.0001 **
		P2	140.17	11.47	104.21	26.26	76.18	3.83	70.94	1.56	-0.57	.001 *	-0.7	.0001 **
		P3	71.48	2.77	72.05	3.55	76.94	2.61	70.54	6.14	0.18	0.121	-0.45	.001 **
		P4	132.41	28.21	72.06	3.55	61.86	2.34	61.01	1.15	-0.8	.0001 **	-0.12	0.214
		P5	98.37	8.46	126.81	14.97	104.27	1.5	67.98	3.65	0.73	.0001 **	-0.86	.0001 **
		P6	71.48	2.77	60.04	2.51	57.89	2.18	60.7	4.29	-0.8	.0001 **	0.3	.016 *
	S1	P1	90.81	3.21	87.35	2.78	69.5	5.4	69.5	5.4	-0.42	.002 *	-0.82	.0001 **
		P2	57.83	0.61	57.7	0.52	54.1	1.58	57.79	8.89	-0.04	0.4	-0.1	0.25
		P3	86.68	15.25	115.81	9.84	95.22	10.48	88	7.39	0.75	.0001 **	-0.32	0.004
		P4	64.07	3.11	67.03	9.14	60.25	0.43	61.42	1.34	0.03	0.424	0.41	.0001 **
		P5	62.43	2.25	59.11	0.93	67.8	5.92	77.907	0.59	-0.73	.0001 **	0.59	.0001 **
		P6	161.89	5.64	170.15	10.64	85.13	16.98	77.91	9.27	0.39	.004 *	-0.1	0.233
	S6	P1	68.72	1.94	69.33	2.23	67.54	8.22	71.63	5.34	0.14	0.183	0.23	.034 *
		P2	57.37	0.3	58.69	3.34	58.62	2.54	60.44	1.18	0.55	.003 *	0.33	.007 *
		P3	139.11	10.22	77.26	8.53	90.61	19.05	101.36	8.99	-0.78	.0001 **	0.45	0.002
		P4	75.88	2.4	75.1	8.92	64.61	4.3	72.11	9.79	-0.04	0.438	0.43	.001 *
		P5	113.15	8.7	110.79	47.13	67.76	9.47	89.36	23.45	-0.17	0.124	0.49	.0001 **
		P6	177.04	13.27	101.91	16.53	145.66	7.16	126.67	11.51	-0.78	.0001 **	-0.68	.0001 **
EDA ^2^	Control	P1	0.189	0.01	0.224	0.011	0.28	0.004	0.31	0.004	0.79	.0001 **	0.87	.0001 **
		P2	0.091	0.002	0.448	0.531	0.499	0.049	0.597	0.103	0.46	.002 *	0.51	.0001 **
		P3	0.003	0.002	0.448	0.531	0.061	0.006	0.091	0.005	0.8	.0001 **	0.85	.0001 **
		P4	0.334	0.47	0.214	0.037	0.216	0.014	0.319	0.049	0.24	.049 *	0.8	.0001 **
		P5	0.0314	0.001	0.0315	0.001	0.04	0.001	0.044	0.002	0.02	0.456	0.74	.0001 **
		P6	0.081	0.001	0.085	0.002	0.108	0.001	0.126	0.002	0.8	.0001 **	0.86	.0001 **
	S 1	P1	0.202	0.055	0.264	0.008	0.328	0.006	0.323	0.004	0.47	.0001 **	-0.41	.0001 **
		P2	0.347	0.005	0.371	0.039	1.468	0.213	1.447	0.279	0.09	0.317	0.24	0.101
		P3	0.064	0.016	0.087	0.017	0.041	0.001	1.447	0.279	0.55	.0001 **	0.86	.0001 **
		P4	0.265	0.004	0.278	0.009	0.189	0.009	1.447	0.279	0.68	.0001 **	0.86	.0001 **
		P5	0.105	0.001	0.108	0.002	0.136	0.002	1.447	0.279	0.74	.0001 **	0.86	.0001 **
		P6	0.182	0.001	0.186	0.002	0.228	0.001	1.447	0.279	0.77	.0001 **	0.86	.0001 **
	S 6	P1	0.265	0.007	0.264	0.007	0.261	0.029	0.202	0.055	0.46	.0001 **	-0.58	.0001 *
		P2	0.319	0.008	0.334	0.009	0.74	0.306	1.22	1.22	0.6	.0001 **	0.83	.0001 **
		P3	0.087	0.027	0.126	0.008	0.08	0.009	0.1	0.034	0.68	.0001 **	0.27	.039 *
		P4	0.328	0.088	0.495	0.147	0.722	0.18	0.903	0.106	0.58	.0001 **	0.56	.0001 **
		P5	0.066	0.002	0.0373	0.018	0.0393	0.01	0.031	0.006	-0.42	.002 *	-0.31	.0001 **
		P6	0.019	0.0003	0.021	0.006	0.061	0.004	0.077	0.006	0.4	0.007	0.86	.0001 **
ACC ^3^	Control	P1	0.988	0.001	0.991	0.002	0.984	0.002	0.987	0.003	0.47	.0001 **	0.41	.001 **
		P2	0.983	0.002	0.986	0.005	0.985	0.001	0.983	0.001	0.21	0.097	-0.68	.0001 **
		P3	0.991	0.003	0.992	0.003	1	0.004	0.993	0.002	0.04	0.399	-0.78	.0001 **
		P4	0.971	0.003	0.972	0.003	0.972	0.004	0.972	0.002	0.18	0.116	0.15	0.149
		P5	0.97	8.882	0.975	0.008	0.984	0.001	0.98	0.003	0.37	0.007	-0.6	.0001 **
		P6	0.986	0.002	0.982	0.002	0.985	0.002	0.987	0.003	-0.66	.0001 **	0.27	.041 *
	S1	P1	0.984	0.003	0.987	0.001	0.992	0.007	0.991	0.001	0.45	0.001	0.15	0.145
		P2	1.01	0.003	1.009	0.002	1.006	0.003	1.008	0.001	-0.12	0.217	0.24	0.056
		P3	0.999	0.012	0.999	0.004	1.015	0.013	1.008	0.016	0.29	.028 *	-0.32	0.012
		P4	N/A	N/A	N/A	N/A	N/A	N/A	N/A	N/A	N/A	N/A	N/A	N/A
		P5	0.984	0.003	0.98	0.006	0.984	0.003	0.986	0.006	-0.31	.019 *	0.23	0.054
		P6	0.995	0.001	0.994	0.002	1.011	0.002	1.007	0.006	-0.3	.037 *	-0.33	.014 *
	S6	P1	0.991	0.002	0.989	0.002	0.996	0.996	1	0.001	-0.33	.019 *	0.15	0.183
		P2	1.006	0.003	1.008	0.001	0.987	0.006	0.99	0.006	0.24	0.056	0.14	0.165
		P3	1.002	0.002	0.9979	0.001	1.0449	0.028	0.9997	0.001	-0.7	.0001 **	-0.81	.0001 **
		P4	1.014	0.007	1.017	0.006	1.045	0.028	1.002	0.004	0.28	.033 *	-0.72	.0001 **
		P5	0.983	0.003	0.984	0.003	1.011	0.008	0.987	0.004	0.07	0.33	-0.86	.0001 **
		P6	1.005	0.01	0.993	0.015	0.993	0.003	0.9952	0.002	-0.4	0.005	0.32	.015 *
ST ^4^	Control	P1	30.08	0.05	31.52	6.66	30.2	0.02	30.07	0.03	0.79	.0001 **	-0.86	.0001 **
		P2	30.87	0.01	30.93	0.07	31.479	0.032	31.526	0.016	0.29	0.027	0.87	.0001 **
		P3	29.25	0.05	29.26	0.11	29.42	0.16	30	0.2	-0.05	0.392	0.85	.0001 **
		P4	28.56	1.58	30.77	0.12	30.72	0.11	31.38	0.05	0.78	.0001 **	0.86	.0001 **
		P5	27.23	0.03	27.41	0.03	27.26	0.01	27.41	0.05	0.8	.0001 **	0.86	.0001 **
		P6	27.86	0.01	27.88	0.01	27.88	0.005	27.919	0.013	0.62	.001 **	0.87	.0001 *
	S1	P1	33.41	0.01	33.32	0.04	32.85	0.03	32.8	0.01	-0.78	.0001 **	-0.77	.0001 **
		P2	34.05	0.03	34.27	0.06	33.856	0.048	33.845	0.029	0.78	.0001 **	-0.17	0.125
		P3	30.52	0.1	29.94	0.35	30.4	0.14	30.29	0.09	-0.78	.0001 **	-0.43	.0001 **
		P4	31.17	0.03	31.21	0.06	31.1	0.04	31.19	0.05	0.3	0.094	0.74	.0001 **
		P5	30.01	0.06	29.96	0.05	29.56	0.07	29.48	0.07	-0.33	.025 *	-0.47	.0001 **
		P6	29.71	0.02	29.82	0.03	30.28	0.044	30.224	0.013	0.78	.0001 **	-0.75	.0001 **
	S6	P1	32.67	0.01	32.73	0.01	32.88	0.05	32.97	0.02	0.78	.0001 **	0.75	.0001 **
		P2	32.92	0.07	32.73	0.01	32.945	0.07	33.116	0.042	-0.78	.0001 **	0.86	.0001 **
		P3	31.25	0.3	31.548	0.14	31.242	0.13	31.562	0.22	0.49	.0001 **	0.76	.0001 **
		P4	33.91	0.06	33.89	0.08	33.82	0.09	33.92	0.06	-0.24	0.066	0.5	.0001 **
		P5	30.91	0.15	31.35	0.36	31.96	0.05	32.17	0.09	0.46	.0001 **	0.81	.0001 **
		P6	27.57	0.28	27.34	0.41	27.63	0.02	27.65	0.02	-0.32	.021 *	0.43	.001 *

^1^heart rate, ^2^electrodermal activity, ^3^movement, ^4^skin temperature. Colour code: dark colours indicate significant differences after Bonferroni correction ( **p<0.0007); pale colours indicate uncorrected standard threshold (*p<0.05). Green = higher during first song than baseline; red = lower during first song than baseline; blue = higher during slow than fast music; orange = lower during slow than fast music.

H5 was not supported by changes in HR. Instead, more participants showed a significantly lower HR during the first song, particularly in the control session. This may reflect the relaxing impact of the music, particularly during the control which is less interactive. There were few significant differences in movement. There were conflicting responses that raised questions about how the physiological measures relate to each other; for example the EDA of P1S2 was significantly higher across all three sessions, whilst ST was significantly higher in the control and session 6 but significantly lower in session 1.

More physiological differences were present in the control session than the intervention sessions. In support of H5, EDA, ST and movement were all significantly higher during the first song of the control session, however HR was significantly lower. During the intervention sessions, only a significant increase in EDA during the first song of both intervention sessions and ST during session 6 were consistent with H5. ST was significantly lower during the first song of session 1.

**Physiological changes related to musical style.** Physiological responses were often significantly different during different styles of music in support of H6. However, there were mixed results regarding which response was higher during which style of music. Overall, there were more significantly higher responses during slow music than fast. EDA was significantly higher during the slow music for five participants in the control session, four in the first session and three participants in sessions 6. EDA was only robustly significantly higher during the faster music in one instance. Although ST was higher during the slow music for four participants in the control session and five in session six, ST was also higher during the fast music for four participants in session 1. HR results were mixed, with little differences found in HR in either intervention session. There were also fewer differences in movement, however there were more instances of significantly more movement during fast music than slow. There were more significant differences in music during the control session, perhaps due to the lack of other variables that may affect physiological responses, such as interaction and instruments.

**Engagement**. Engagement was higher in the intervention sessions than the control session, which is reflective of the interactive nature of the sessions ( Table 10). Although all three participants were more engaged in session 6 than the control session, only P1 and P3 showed an increase in engagement in session 1 compared to control. Physiological measures were not consistently related to engagement for any of the participants. P2S2 showed significant differences between measures during fast and slow music but no difference in engagement as the participant remained still throughout. This highlights that an individual may be experiencing more than appears visible to an observer.

**Table 10.** Engagement during control and intervention sessions.

Metric	ID	Control		Session 1		Session 6	
		Fast	Slow	Fast	Slow	Fast	Slow
Song length	P1	225s	252s	335s	214s	214s	255s
	P2	225s	252s	335s	214s	214s	255s
	P3	225s	252s	335s	214s	214s	255s
Positive engagement	P1	220s (17%)	1512s (23%)	2010s (20%)	453s (35%)	418s (33%)	510 (33%)
	P2	0 (0%)	0 (0%)	0 (0%)	0 (0%)	307 (24%)	288 (19%)
	P3	317 (23%)	279 (18%)	675 (34%)	454 (35%)	446 (35%)	462 (30%)
Negative engagement	P1	0(0%)	0 (0%)	0 (0%)	0 (0%)	0 (0%)	0 (0%)
	P2	0 (0%)	0 (0%)	0 (0%)	0 (0%)	0 (0%)	0 (0%)
	P3	0 (0%)	0 (0%)	0 (0%)	0 (0%)	0 (0%)	0 (0%)

**Figure 3.**
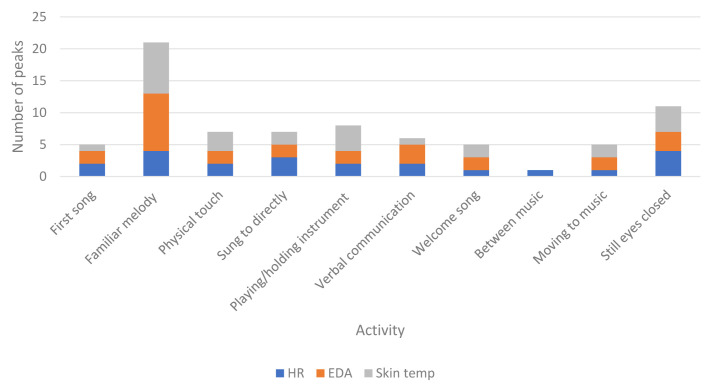
Activity during peaks in physiological data for all participants in all sessions.

***Peaks in the data.*** H8 stated that peaks in physiological responses will be associated with visible engagement. Times that physiological responses were highest across the whole of each session for each participant were identified and matched to the video footage to observe what was occurring at these specific times. Specific activities occurring during peaks in physiological responses across all participants are described in Table 11. Figure 3 shows peaks during different activities throughout the sessions.

**Table 11.** Activity during peaks in physiological responses of all participants in study 2.

	Control	Session 1	Session 6
P1			
Heart rate	Touching hand of staff on her lap. Sits forward in chair, taps foot, gentle music playing	Facilitator uses participants name and hands her an instrument	Same melody as first song, flute is being played next to her
EDA	Just before increase highest skin temp scores, final song of the session, tapping leg	Being directly sung to as part of the welcome song by two facilitators	Towards end of welcome song, staff member is holding hand and swaying
ST	The final melody playing (similar to first song), rubbing leg, leans to speak to staff	First song, sitting very still but visually alert, turns head to watch facilitator play flute	Leaning forward, holding instrument, tapping hand, same melody as first song
P2			
Heart rate	One minute in to the first piece of music being played. Visually alert looking around	Sitting still, facilitator is next to him playing a xylophone, sharp noise, no visual response	Shaking an instrument intently with support from facilitator
EDA	Towards the end of the first song, visually alert, looking around the room	During the second half of the welcome song	Playing a percussion instrument with a beater, clarinet and harp being played near
ST	Beginning of final song which is similar melody to first song. Sitting very still in chair	Facilitator is next to him singing the welcome song	During final song, same melody as first song, sitting still, opens his eyes intermittently
P3			
Heart rate	Energetic song (second to last) had just finished, had been tapping her feet, starts speaking to facilitator	Towards the end of the first song, tapping foot visually alert	Handed an instrument for the first time in session, tapping foot and using instrument, appears alert
EDA	About 2/3 through the session, very alert, tapping foot leans forward in chair and speaks to facilitator	When the first song melody is played again at the end of the session	Being sung to directly including her name ‘young at heart’, flute played in front of her
ST	Start of the final song which has a similar melody to the first song sitting still	Between songs holding an instrument up, visually alert	Being sung to directly including her name ‘young at heart’, flute played in front of her
P4			
Heart rate	First song	Tapping foot and hand, flute played next to him	Welcome song, sung to directly
EDA	Tapping foot to music, same song as ST peak but later in song	Last song same melody as first song, tapping foot	Final song, tapping foot, familiar melody, drum nearby
ST	Tapping foot to fast music, visually alert	Holding instrument, flute nearby	Welcome song, tapping foot to music, sung to
P5			
Heart rate	Final song, same melody as first	Visually alert, holding instrument in lap, flute nearby	First song, familiar melody, eyes closed
EDA	Final song, same melody as first	Leaning forward holding instrument	Final song, familiar melody, looking around
ST	Still, faster music starts, staff hand on arm	First song, familiar melody	Final song, familiar melody, looking around
P6			
Heart rate	Final song, same melody as first	First song, familiar melody	Sitting still, eyes open, flute playing nearby
EDA	Final song, same melody as first	Last song, familiar melody	Final song, familiar melody, touch by staff
ST	Sitting still, percussion improvisation	Last song, familiar melody, staff holding hand	Final song, familiar melody holding instrument

* ‘First song’ relates to the first song in the control session which is then repeated at the beginning and the end of each session and thereafter recorded as a ‘familiar melody’

## Discussion

### Study 1

H1 predicted an increase in physiological responses during the first song of the session compared to before the session began, which was partially supported by changes in EDA and HR. All participants showed a significant increase in EDA during the first song and this was robustly significant for six of eight participants. Although EDA has been linked to different emotions associated with arousal including anticipatory excitement and fear ( Kreibig, 2010), the experience was likely to be positive in this instance considering continued voluntary participation of group members and verbal comments they made after the session. The increase in HR during the first song may be indicative of excitement ( Wilhelm *et al.*, 2006) and/or a reflection of the energy required to sing ( Bernardi *et al.* 2017).

Consistent with H2, physiological responses differed during different styles of music. In line with previous research which found increased physiological arousal in response to faster tempo music ( Bernardi *et al.*, 2006; Gomez & Danuser, 2007), HR and ST were significantly higher for more participants during energetic, faster music than during walking pace music. The high number of significant EDA results relating to different music styles is reflective of previous research which found increased EDA during emotional responses to music ( Gomez & Danuser, 2004).

### Study 2

***Comparisons of physiological responses between sessions.*** There was an overall increase in EDA and movement during the first song of the intervention sessions compared to the control, supporting H3. Engagement was also higher during the fast and slow music of the intervention sessions compared to control. Previous research suggests that an increase in movement may indicate increased engagement ( Perugia *et al.*, 2018) and reduced depression or apathy ( David *et al.*, 2010). Increased EDA whilst listening to music in healthy adults has been linked to pleasure ( Salimpoor *et al.*, 2009); it is possible that the introduction of live instruments enhanced interest and enjoyment. The increase in engagement is reflective of the interactive nature of the intervention sessions, during which participants are encouraged to play instruments. Perhaps reflective of previous findings that HR is difficult to interpret due variety of potentially influential factors ( Wilhelm *et al.*, 2006), the HR results were mixed and did not support H3.

Overall, physiological responses were significantly higher during the first song of session 1 than session 6, therefore H4 was not supported. This was particularly evident in HR and EDA, suggesting physiological responses diminish as the intervention becomes less novel, or participants may have become more comfortable with the group and process ( Clare *et al.*, 2020). There were fewer differences in movement, which may be expected as both intervention sessions encourage interaction.

***Physiological responses within sessions.*** EDA and ST were higher overall during the first song than baseline. This was particularly evident in EDA which is reflective of study 1 and associated with increased pleasure ( Salimpoor *et al.*, 2009). Increases in ST have been associated with music eliciting calm and positive emotions in healthy adults ( McFarland, 1985). EDA increased during the control session for four participants, suggesting listening to music alone is also beneficial, however there were more significantly robust increases in EDA during the intervention sessions. In contrast to study 1, H5 was not supported by HR, which was often lower during the first song of the session than baseline. A reduction in HR has been related to improved mood ( Raglio *et al.*, 2010) and may be reflective of the relaxing nature of the intervention sessions in contrast to the energy required to sing in Study 1.

Physiological responses were predicted to differ depending on the style of music playing (H6) and this hypothesis was partially supported, however results were inconsistent. Overall, the case studies found more, significantly higher physiological responses during slow music, particularly for EDA and ST to a lesser extent. ST results were not consistent across different sessions. Most participant’s ST was significantly higher during the slow music of the control and session 6, and during the faster music of session 1. This suggests other factors aside from musical style may be having an influence. In contrast to Study 1 and previous research that found an increase in HR during different songs ( Norberg *et al.*, 2003), HR results were inconsistent. There were more significant differences between fast and slow music during the control than the intervention sessions. Musical style may have less influence during the interactive sessions as there are more variables that may have an impact (e.g. whether they were playing an instrument or one-on-one interactions).

H7 proposed that changes in physiological responses will be associated with rated and visible engagement. Engagement is a way of monitoring how helpful an activity is for a PLWD and was described by Perugia *et al.* (2018) as the “psychological state of wellbeing, enjoyment and active involvement that is triggered by meaningful activities” (p 112). There was little difference between rated engagement during different types of music, which was also reflected by mixed physiological responses. Previous research has linked changes in EDA to engagement due to changes during episodes of excitement and attention ( Andreassi, 2007; Perugia *et al.*, 2017). Although physiological responses reflected rated engagement at times, this was not consistent enough to support H7. For example, P2S2 showed a peak in ST, EDA and movement in session 1 despite not visually appearing engaged. This suggests a person may be experiencing more than is visually obvious, which is useful information for encouraging carers to continue to offer interactive activities regardless of whether the PLWD appears unengaged.

***Peaks in the data.*** Activity during peaks in the data partially supported H8. Activities related to visible engagement were present, including physical touch or interacting with an instrument, however the most common activity during the highest physiological responses was the presence of a familiar melody. In line with the notion of “inclusion” ( Kitwood, 1997), responses were also high when participants were being sung to using their name. These findings indicate that individual interactions fostering elements of personhood such as identity/inclusion and occupation (playing instruments) create changes in physiological responses that may be related to enjoyment and stimulation ( McFarland, 1985; Salimpoor *et al.*, 2009). Having a role in creating music may also have met Nolan *et al.* (2004)’s senses of “achievement” and “purpose”.

ST and EDA peaked at similar times, including listening to familiar melodies, physical touch or holding an instrument. Activity during increased HR was more varied, yet also included familiar music and being sung to. In line with findings related to H7, peaks also occurred when participants appeared disengaged with their eyes closed. It may be that although participants were not visually engaged in the sessions, having their eyes closed could be an indication of intense enjoyment rather than disengagement. Listening to music with closed eyes can enhance the experience by limiting visual noise and enabling the individual to focus.

### Strengths and limitations

A multiple-case study design allows analysis of data within and across different case studies ( Chamberlain *et al.*, 2004; Yin, 2003) and evidence formed from studies of this nature has been considered strong and reliable ( Baxter & Jack, 2008). Yin emphasized the importance of four factors; construct validity, internal validity, external validity and reliability ( Yin, 2003) and these factors will be considered below.

Yin suggests construct validity is obtained by multiple sources of evidence, which has been more effectively achieved across both studies through the concurrent measurement of multiple physiological signals, and particularly effectively achieved in study 2 by additionally utilising video footage and an external rater. The use of established SMA to detect patterns in physiological responses enhances the internal validity of this research ( Borckardt & Nash, 2014). Using responses of the ANS can be challenging due to potential external influences such as movement, interactions and enjoyment ( Kim & Andre, 2008) and the high degree of variation between individuals and over time ( Jaimovich *et al.*, 2012). Using video data along with the physiological responses strengthens this research as it has allowed a more detailed understanding of how a person’s presentation may relate to the measures on an individual basis. For example, in addition to providing information about participants appearing engaged when their physiological responses appeared elevated, video footage enabled identification of small gestures that may be having an impact on physiology (e.g. eye contact or physical touch) that otherwise may have been missed.

For each of the measures analysed individually, the statistical analysis used did not correct for covariates which may be considered a threat to internal validity. It is possible that there would be an impact. EDA for example, may be impacted by movement ( Khan *et al.*, 2019). Encouragingly, there was a non-significant difference in movement in some of the conditions where significant changes in EDA were observed, demonstrating the possibility for these distinct physiological changes to occur in isolation.

Case studies are generally considered to have low external validity ( Jacobsen, 2002). Collating multiple case studies may limit the time that can be spent on each individual observation, yet increase representativeness ( Gerring, 2004). The naturalistic setting of this study meant that participants were not randomly selected, and all participants were either white British or white European. These factors in addition to the small number of cases make it difficult to extrapolate findings to a wider population. In study 1, a number of confounding variables may have been accounted for as the group had been running for two months so participants would be familiar with the group and environment, however study 2 was a new intervention and they would have only met the musician-facilitators at the control session. It is therefore difficult to attribute physiological changes to the activity alone and not the novel group setting. However, the inclusion of a control session identified increased physiological changes in the intervention session suggesting the activity was having an impact.

Due to the variability in the data, it would have been beneficial to observe interactions during the lowest points in addition to during the peaks. Without observing behaviour during the troughs for an absence of interactions/familiar music, it is difficult to conclude that the increase in responses is related directly to these events.

### Practice implications for musicians, community organisations, residential care and healthcare professionals

In line with previous research (e.g. Livingston *et al.*, 2014) this research indicates that music-based activities are beneficial for people with dementia, as there were increases in physiological responses associated with enjoyment and engagement. Although these outcomes should be considered tentatively due to the methodological limitations, there is a good deal of research that supports the efficacy of music and singing in dementia (e.g. Cho, 2018; Jacobsen *et al.*, 2015; Särkämö *et al.*, 2014; Unadkat *et al.*, 2017). Peaks in the physiological data when an individual does not appear engaged, highlight that visible observations alone may not provide the whole picture. As previously stated, having closed eyes may be an indication that a person is attending to the music more intently. These physiological increases therefore emphasise the potential benefits of activities even when a visible indication of engagement does not appear obvious. Incorporating elements of the group that were in line with Kitwood’s principles (e.g. “inclusion”, by encouraging singing directly) appeared to lead to increases in physiological response. Non-intrusive physiological measurement may be a beneficial way of gathering more information about the most engaging aspects of an activity and inform the development of future interventions. While further research is needed “to differentiate the role of music across different types of dementia and for different groups of individuals” ( Bowell & Bamford, 2018, p. 16), residential care settings and community organisations can feel confident that music and singing activities provide benefits for this population across levels of impairment, and healthcare professionals should consider recommending music and singing groups as part of dementia care.

### Future research

Differences between intervention sessions suggest that following a community group longitudinally may be beneficial to observe changes in physiological responses over time, or establish better estimates of the magnitude and quality of impact such sessions have when participants are able to participate in sessions regularly. Future multiple-case study research should place emphasis on construct validity ( Yin, 2003) by collating physiological measures alongside video analysis, observations and psychometric measures when appropriate. This may provide a clearer understanding of what physiological responses may be telling us and what wellbeing and engagement mean for this population (e.g. Strohmaier *et al.*, 2021). As peaks in physiological data were associated with familiar music and playing instruments, consideration of the participant’s prior musical interests and relationship with singing/playing an instrument earlier in life should be noted in future research. It is easy to recommend that larger sample sizes will provide additional information, but alongside this, looking specifically at how physiological and behavioural responses vary according to type and severity of dementia will also help to further develop dementia care strategies and tailor interventions.

## Conclusions

The aim of these two linked multiple-case studies was to observe physiological responses of people at different stages of dementia during two music-based activities. During a community singing group, EDA and HR increased indicating increased arousal and enjoyment. HR and ST were higher during faster music and EDA was influenced by different musical styles. During an interactive music group, EDA, movement and rated engagement were all higher compared to the control session (music listening). When compared to baseline, EDA and ST were higher and HR was lower during the intervention suggesting a calming, emotional response. Physiological responses peaked during familiar music, personal interactions and physical touch. Peaks also occurred at times when that the individual appeared disengaged. These case studies indicate that music-based activities may increase arousal and/or engagement for people living with dementia. Future research of physiological measures longitudinally and in conjunction with video-analysis and/or psychometric measures will enrich our understanding of how engagement and wellbeing interact for this population.

## Data availability

### Underlying data

Zenodo: Underlying dataset for the study: Singing and music making: Physiological responses across early to later stages of dementia. http://doi.org/10.5281/zenodo.4704417 ( Walker *et al.*, 2021a).

This project contains the following underlying data:

- Walker *et al*_Study 1 Physiological Data.xlsx (Physiological data for Study 1)
- Walker *et al*_Study 2 Physiological Data.xlsx (Physiological data for Study 2)
- Walker *et al*_Video coding data_VD-10E scores_Study 2.xlsx (Video Coding – Incorporating Observed Emotion (VC-10E) engagement scores in seconds. Scores measuring engagement across three sessions in Study 2)

Data are available under the terms of the Creative Commons Attribution 4.0 International license (CC-BY 4.0).

### Extended data

Zenodo: Singing and music making: Physiological responses across early to later stages of dementia extended files. http://doi.org/10.5281/zenodo.4704596 ( Walker *et al.*, 2021b).

This project contains the following extended data:

- Extended File_Walker *et al.*, 2021.docx and .pdf (For Study 1, figures depicting results for HR, EDA, ACC and ST for P1S1 before the session starts, during the first song and during fast and slow music. This is repeated for each of the nine participants. For Study 2, HR, EDA, ACC and ST for P1S2 before the session starts, during the first song and during fast and slow music. Each figure includes the physiological measures during the control and both intervention sessions. This is repeated for each of the six participants.)

Data are available under the terms of the Creative Commons Attribution 4.0 International license (CC-BY 4.0).
