# Supplementary material for: Singing and music making: physiological responses across early to later stages of dementia
Source: Wellcome Open Res. 2022 Feb 14;6:150. Originally published 2021 Jun 14. [Version 3] doi: 10.12688/wellcomeopenres.16856.3 (PMC8864187; doi:10.12688/wellcomeopenres.16856.3)
Supplement: Peer review history

# Peer review: Reviewer response for version 3

**DOI:** 10.21956/wellcomeopenres.19602.r48690

**Published:** 2022-02-22

**Authors:** Loris Tamara Schiaratura

**Affiliations:** 1 PSITEC Lab, Faculty of Psychology and Educational Sciences, University of Lille, Lille, France

I don't have new comments

Is the work clearly and accurately presented and does it cite the current literature?

Yes

If applicable, is the statistical analysis and its interpretation appropriate?

Yes

Are all the source data underlying the results available to ensure full reproducibility?

Yes

Is the study design appropriate and is the work technically sound?

Yes

Are the conclusions drawn adequately supported by the results?

Partly

Are sufficient details of methods and analysis provided to allow replication by others?

Yes

Reviewer Expertise:

Social psychology; Nonverbal Communication in Alzheimer disease;

I confirm that I have read this submission and believe that I have an appropriate level of expertise to confirm that it is of an acceptable scientific standard.

# Peer review: Reviewer response for version 2

**DOI:** 10.21956/wellcomeopenres.19098.r47856

**Published:** 2022-01-28

**Authors:** Loris Tamara Schiaratura

**Affiliations:** 1 PSITEC Lab, Faculty of Psychology and Educational Sciences, University of Lille, Lille, France

This research consists of 2 studies that aim to evaluate the effects of musical activities in people with dementia, at different stages of the disease. While the 2 studies are presented as linked, they differ in the musical activities used and the stage of the participants’ disease: Study 1 uses singing in mild moderate dementia while study 2 uses interactive music in advanced dementia.

 Their common interest lies in that they are both taking physiological and movement measures in relation to emotional engagement and visual behavior. It also lies in the multiple case analysis allowing a detailed analysis of the different individual responses and their relationships.

 The underlying objectives of these studies are, in my opinion, presented in a too general way. A clear presentation of the objectives for each study would have allowed for a better understanding of the hypothesis. However, for each study, the methodological descriptions are detailed and clearly presented.

- P2 Abstract. The number of 18 participants is still present (while 9 in study1 and 6 in study 2).
- P6. In study 1: Two songs for the four selected, differing in tempo, were used for comparison. What are the criteria that have been used to determine this choice?
  Il the same study 1, why were the participants intermittently asked to stand and sit down?
- P7. In study 2: Engagement was assessed for only three participants (Two male and one female). More precisely, what do you mean by “Participants were selected that were visibly different in their engagement”.
- There are numerous results and deep analysis (especially for study 2). The organization based on each hypothesis and on each physiological outcome promotes a good understanding of the relationships between them.
- P10 and 17: The titles for tables 8 and 10 are to be verified. In addition, the presentation of data for engagement could be more uniform (in seconds and/or time percentages).
- In the discussion, the disease stages (moderate and advanced) that distinguish the studies are not taken well enough when discussing the differences in physiological data between these two studies. More specifically in study 2, visible engagement is not be associated with physiological responses.  It could be argued that emotional disorders (e.g. depression) and deficits in motor abilities could also play a role (e.g. Pongan, *et al.*, 2017). ^1^ But unfortunately, they are not assessed in the research.
- Finally, in relation to the conclusions, it could have been interesting to differentiate socio-emotional engagement and motor engagement in video-analysis (e.g. Hobeika *et al.*, 2021). ^2^

Is the work clearly and accurately presented and does it cite the current literature?

Yes

If applicable, is the statistical analysis and its interpretation appropriate?

Yes

Are all the source data underlying the results available to ensure full reproducibility?

Yes

Is the study design appropriate and is the work technically sound?

Yes

Are the conclusions drawn adequately supported by the results?

Partly

Are sufficient details of methods and analysis provided to allow replication by others?

Yes

Reviewer Expertise:

Social psychology; Nonverbal Communication in Alzheimer disease;

I confirm that I have read this submission and believe that I have an appropriate level of expertise to confirm that it is of an acceptable scientific standard, however I have significant reservations, as outlined above.

## References

[1] : Can Musical or Painting Interventions Improve Chronic Pain, Mood, Quality of Life, and Cognition in Patients with Mild Alzheimer's Disease? Evidence from a Randomized Controlled Trial. *J Alzheimers Dis* .2017;60(2) : 10.3233/JAD-170410 663-677 10.3233/JAD-170410 28922159

[2] : Socio-emotional and motor engagement during musical activities in older adults with major neurocognitive impairment. *Scientific Reports* .2021;11(1) : 10.1038/s41598-021-94686-4 10.1038/s41598-021-94686-4 34315954PMC8316394

# Response

**Published:** 2022-02-07

**Authors:** Paul Camic

**Affiliations:** University College London, UK

Reviewer 2 comments

 Loris Tamara Schiaratura, PSITEC Lab, Faculty of Psychology and Educational Sciences, University of Lille, Lille, France 

 Dear Dr. Schiaratura,

 Thank you for your detailed and encouraging comments. We deeply appreciate the time you took and the effort required to review our article. We have attempted to address your comments below:

 1.    P2 Abstract. The number of 18 participants is still present (while 9 in Study 1 and 6 in Study 2).

 Response: This was unintendedly not changed, and we have now made clear that this reflects 18 data points (6 participants across 3 music conditions) and not 18 participants in study 2. We have also further differentiated Study 1 and Study 2 in the abstract.

 2.    P6. In Study 1: Two songs for the four selected, differing in tempo, were used for comparison. What are the criteria that have been used to determine this choice?

 Il the same study 1, why were the participants intermittently asked to stand and sit down? Response: The criteria to determine the choice of songs was determined by the choral director along with the pianist. The researchers requested that songs differ in tempo and genre. Two songs with the most contrasting temps were selected for comparison. Participants were asked to intermittently stand during different parts of songs in order to assess physical movement on the Empatica device with a clear physical maker (standing up and sitting down), in addition to stretching at the beginning of each session as a physical marker, which was already incorporated into the weekly choral routine; this has been added to the manuscript.  

 3.    P7. In study 2: Engagement was assessed for only three participants (Two male and one female). More precisely, what do you mean by “Participants were selected that were visibly different in their engagement”. Response: We have clarified this in the manuscript: “Participants were selected that were visibly different including two participants that demonstrated more physical movement and one participant appearing disengaged with their eyes closed. Two of the participants were male and one was female.”

 4.    P10 and 17: The titles for tables 8 and 10 are to be verified. In addition, the presentation of data for engagement could be more uniform (in seconds and/or time percentages). Response: Thank you for bringing this to our attention. We have removed one of the tables as they were partially including overlapping data. Table 10 has now been renumbered and labelled as Table 8 with the corresponding Figure 2. 

 5.    In the discussion, the disease stages (moderate and advanced) that distinguish the studies are not taken well enough when discussing the differences in physiological data between these two studies. More specifically in study 2, visible engagement is not be associated with physiological responses.  It could be argued that emotional disorders (e.g. depression) and deficits in motor abilities could also play a role (e.g. Pongan, et al., 2017). But unfortunately, they are not assessed in the research. Response: We have added this to the discussion section: “While it could be argued that emotional problems and motor abilities can play a role in earlier stages of dementia (e.g. Pongan et al., 2017), in the present study there were no self-reported emotional problems or deficits in motor abilities by participants in Study 1. In Study 2 deficits in motor abilities or emotional problems were not noted by care facility staff at the time of recruitment, nor were motor deficits noted by researchers when participants entered and left the room where the activities took place.” 

 6.    Finally, in relation to the conclusions, it could have been interesting to differentiate socio-emotional engagement and motor engagement in video-analysis (e.g. Hobeika et al., 2021). Response: We have now included this in the discussion of Study 2:”… it is possible that the introduction of live performance (Hobeika et al.,2021) and the hands-on use of instruments, enhanced interest and enjoyment.” Also added the following to the strengths and limitations section: “Given the opportunity for detailed video-analysis, it may have been interesting to differentiate socio-emotional engagement (e.g. gaze direction/facial expressions) from motor engagement (e.g. overall body movement) as in previous research (Hobeika et al., 2021).” 

 Thank you.

 Sincerely,

 Paul Camic on behalf of the other co-authors

# Peer review: Reviewer response for version 2

**DOI:** 10.21956/wellcomeopenres.19098.r46641

**Published:** 2021-11-08

**Authors:** Jeanette Tamplin

**Affiliations:** 1 Melbourne Conservatorium of Music, Faculty of Fine Arts and Music, The University of Melbourne, Melbourne, VIC, Australia

The revised paper has addressed most of the issues raised in my previous review and the paper is stronger as a result. There is just one remaining issue regarding the reference to 18 *participants* in the abstract. Please revise this to 18 data points (or something similar) for 6 participants.

 There are 2 typographical errors also: p5 last paragraph - " *convivence sample*" should be " *convenience sample*" I think. p7 - the acronym for Video Coing - Incorporating Observed Emotion should have letters not numerals (ie. *VC-IOE* not *VC-10E*).

 Consider changing the order of these 2 sentences on p7 to improve flow of information: " *Participants were selected that were visibly different in their level of engagement and included two male and one female. Due to the highly detailed and time intensive, second-by-second video analysis, engagement was assessed for only three participants.*"

 This paper offers a great contribution to the research literature exploring the effects of music for people living with dementia.

Is the work clearly and accurately presented and does it cite the current literature?

Yes

If applicable, is the statistical analysis and its interpretation appropriate?

I cannot comment. A qualified statistician is required.

Are all the source data underlying the results available to ensure full reproducibility?

Yes

Is the study design appropriate and is the work technically sound?

Yes

Are the conclusions drawn adequately supported by the results?

Partly

Are sufficient details of methods and analysis provided to allow replication by others?

Yes

Reviewer Expertise:

Music therapy in neurological and neurodegenerative conditions

I confirm that I have read this submission and believe that I have an appropriate level of expertise to confirm that it is of an acceptable scientific standard.

# Response

**Published:** 2021-11-08

**Authors:** Paul Camic

**Affiliations:** University College London, UK

Thank you again for reviewing our revised article and for these additional helpful suggestions. We agree, and will add them to a revised version once the second review has been responded to.

# Peer review: Reviewer response for version 1

**DOI:** 10.21956/wellcomeopenres.18594.r45353

**Published:** 2021-08-23

**Authors:** Jeanette Tamplin

**Affiliations:** 1 Melbourne Conservatorium of Music, Faculty of Fine Arts and Music, The University of Melbourne, Melbourne, VIC, Australia

This is an interesting and useful contribution to the literature on the physiological effects of music participation for people living with dementia at different stages of disease progression. The 2 studies are clearly linked and build from one another, however the multiple case study design used in both studies, makes the presentation of results quite complex and difficult to digest. I wonder if it might be better to present the results of the 2 studies in separate publications?

 There are a lot of abbreviations in the paper, which makes it difficult to follow. Please reduce where at all possible. Eg. HRV only appears once and therefore does not need to be abbreviated.

**Abstract:**

 I’m not sure where the 18 participants come from (eg. 14/18, 10/18, 9/18, 13/18) when there were only 9 participants in study 1 and 6 participants in study 2. Perhaps it refers to the 3 music conditions (control/music listening, session 1 and session 6) x 6 participant in study 2? If so, this needs to be explained.

 Abstract conclusion refers to “participating in arts and other activities”, but should be more specific about music participation as per the study described.

**Page 3:**

 en summarising the Van der Steen et al Cochrane results, rather than stating that music-based activities have “little or no impact” on agitation or emotional wellbeing, it might be more accurate to say “little or no evidence of impact”.

 Page 3 – Barrett reference repeats the word “claim” = “…claimed that it is not possible to claim”

 Page 3 – last paragraph – “music has the capacity to ***influence*** emotions”

 The rationale for study 1 and study 2 and description of methodology are mostly clear.

 Why did you only rate engagement for 3 of the 6 participants in study 2?

**Results **– check for consistent use of past tense.

 Also, check that you are referring to the ‘pace’ of music rather than the ‘style’ of music when comparing outcomes for fast vs slow paced songs.

 Because there are numerous physiological outcomes covered in your hypotheses and multiple cases rather than means, this makes it difficult to clearly accept or reject your hypotheses. Perhaps it would it be clearer to have a different hypothesis for each outcome?

 On p12, when you say that “All participants showed an increase in engagement…” do you mean all 3 participants for whom engagement was rated?

 P13, Summary of physiological changes – end of paragraph seems overstated as based on only 1 participant

 Avoid interpretation of results in the results section. Save this for the discussion (eg. This may reflect the relaxing impact of the music).

 When you say: “In support of H5, EDA, ST and movement were all significantly higher during the first song of the control session”, do you mean for all participants?

 “Physiological changes related to musical style” would more accurately be described as “Physiological changes related to musical pace”

**P17:** what do you mean by: “There were more significant differences in music during the control session”? Are you referring to differences in physiological responses to the music?

 “P2S2 showed significant differences between measures?” Do you mean “physiological measures” here? Please be specific.

**P19:**

 words missing in paragraph 1: “HR is difficult to interpret due (to a) variety of potentially influential factors.”

 It would be good to see some further discussion of the results presented in Figure 3, e.g. The activity peaks in physiological data during “familiar melody” – this could also be linked to other literature.

 It was very interesting and important to discuss the finding of physiological arousal during music even when participants with dementia had their eyes closed and appeared not to be engaged.

Is the work clearly and accurately presented and does it cite the current literature?

Yes

If applicable, is the statistical analysis and its interpretation appropriate?

I cannot comment. A qualified statistician is required.

Are all the source data underlying the results available to ensure full reproducibility?

Yes

Is the study design appropriate and is the work technically sound?

Yes

Are the conclusions drawn adequately supported by the results?

Partly

Are sufficient details of methods and analysis provided to allow replication by others?

Yes

Reviewer Expertise:

Music therapy in neurological and neurodegenerative conditions

I confirm that I have read this submission and believe that I have an appropriate level of expertise to confirm that it is of an acceptable scientific standard, however I have significant reservations, as outlined above.

# Response

**Published:** 2021-10-11

**Authors:** Paul Camic

**Affiliations:** University College London, UK

Response to reviewer 1 (Dr. Jeanette Tamplin)

 Thank you for your close reading of our research and for your helpful comments; they are very much appreciated. We agree that both studies discussed in the present article are complex but feel that they are importantly linked, hence the decision to incorporate them within one paper. Within the text we have amended or made changes according to your suggestions (e.g. consistent tense, participant numbers, grammatical consistency, removing a potentially overstated change regarding one participant, etc.). We carefully considered your suggestion to have a different hypothesis for each outcome but believe that its present organisation, although admittedly dense, better conveys the physiological measures.  

 Regarding our use of the term “musical style”: Thank you for addressing this and we agree, it is not precise nor does it indicate speed. Thank you also for your suggestion about using the term “pace” but on reflection, we believe the term “tempo” best describes the different musical pieces we used in the research. We have also noted a change from ‘Style’ to ‘Genre and Tempo’ in Table 1. Your comment was particularly helpful to us in that we also added to the Discussion the importance of considering tempo along with genre in that the same genre has different tempo possibilities, and this is something to explore in future research. 

 We have also added a brief discussion point regarding physiological peaks and a reference to “familial melody”: “Previous research demonstrated that memory for music may be retained longer than other information (Cuddy & Duffin, 2005). In this case, the melodies would only have been recognised from earlier, in the session/intervention therefore increased physiological responses may be suggestive of some ability to hold the melody in short term memory.”

 Kind regards,

 Paul Camic, on behalf of the authors
